# Purification, crystallization and preliminary X-ray diffraction analysis of the 23S rRNA methyltransferase RlmJ from *Escherichia coli*

**DOI:** 10.1107/S1744309113020289

**Published:** 2013-08-19

**Authors:** Avinash S. Punekar, Maria Selmer

**Affiliations:** a Department of Cell and Molecular Biology, Uppsala University, PO Box 596, 751 24 Uppsala, Sweden

**Keywords:** RlmJ, *S*-adenosylmethionine, methyltransferases, 23S rRNA, m^6^A2030, ribosome assembly, *Escherichia coli*

## Abstract

Methyltransferase RlmJ uses the cofactor *S*-adenosylmethionine to methylate the exocyclic nitrogen N6 of nucleotide A2030 in 23S rRNA during ribosome assembly in *Escherichia coli*. RlmJ with a C-terminal hexahistidine tag was overexpressed in *E. coli* and purified as a monomer using Ni^2+^-affinity and size-exclusion chromatography. The recombinant RlmJ was crystallized using the sitting-drop vapour-diffusion method and a full data set was collected to 1.85 Å resolution from a single apo crystal. The crystals belonged to space group *P*2_1_, with unit-cell parameters *a* = 46.9, *b* = 77.8, *c* = 82.5 Å, β = 104°. Data analysis suggested two molecules per asymmetric unit and a Matthews coefficient of 2.20 Å^3^ Da^−1^.

## Abstract 2

The 23S rRNA methyltransferase RlmJ from *E. coli* has been cloned, expressed, purified and crystallized. X-ray diffraction data to 1.85 Å resolution have been collected from the apo RlmJ crystals.

## 1. Introduction

Ribosome biogenesis in all species involves post-transcriptional modification of ribosomal RNA. The functional roles of most modifications remain unknown, but many of them cluster in functionally important regions of the ribosome. In bacteria, these modifications are added by site-specific enzymes and in *Escherichia coli* the most frequent types are methylations of the N, O or C atoms of nucleotides (Sergiev *et al.*, 2011 ▶). The *yhiR* gene product (UniProt ID P37634) was recently identified as the *S*-adenosylmethionine-dependent methyltransferase (MTase) responsible for methylation of the exocyclic nitrogen N6 of adenosine 2030 (m^6^A2030) in the 23S rRNA of *E. coli* and, in accordance with other large-subunit rRNA MTases, was renamed RlmJ (Golovina *et al.*, 2012 ▶). The target nucleotide A2030 is located close to the peptidyl-transferase centre (PTC) in domain V of 23S rRNA. MTase activity of RlmJ was observed on deproteinized 23S rRNA, but not on completely assembled 50S subunits (Golovina *et al.*, 2012 ▶), agreeing with observations that RlmJ modifies A2030 at an early stage of 50S subunit assembly (Siibak & Remme, 2010 ▶). A *ScanProsite* (de Castro *et al.*, 2006 ▶) analysis of the RlmJ sequence had previously identified the N6 adenine-specific DNA methylase signature (Palchevskiy & Finkel, 2006 ▶). With the aim of clarifying how RlmJ recognizes its target and catalyses methyl transfer, we have started a crystallographic structure-determination project.

In this study, we describe the cloning, expression, purification and crystallization of RlmJ.

## 2. Materials and methods

### 2.1. Cloning, expression and purification

The *yhiR* gene was amplified from *E. coli* K-12 MG1655 genomic DNA using polymerase chain reaction (PCR) primers FP1 and RP1 (Table 1 ▶). The PCR product was used as a template for a second PCR using primers FP2 and RP2 (Table 1 ▶) and the product was cloned into pEXP5-CT/TOPO (Invitrogen). The resulting plasmid pAP01-rlmJ encoding recombinant RlmJ [(RlmJ residues 1–280)–(linker SKG)–(hexahistidine tag HHHHHH)] was verified by sequencing and transformed into *E. coli* BL21(DE3) cells. The transformed cells were grown in 500 ml LB medium (100 µg ml^−1^ ampicillin) at 310 K until the OD_600_ ≃ 0.5. Expression at 289 K overnight was induced by the addition of 0.5 m*M* isopropyl β-d-1-thiogalactopyranoside.

All purification steps were performed at room temperature. The cell pellet was resuspended in buffer *A* (20 m*M* Tris–H_2_SO_4_ pH 7.5, 150 m*M* Na_2_SO_4_, 5% glycerol, 5 m*M* β-mercaptoethanol) supplemented with 0.1% Triton X-100, 5 mg lysozyme and Complete protease inhibitor (Roche, Germany) and lysed using a cell disruptor (Constant Systems Ltd, UK). The lysate was clarified by centrifugation, passed through a 0.2 µm filter and loaded onto a Bio-Rad Econo-Pac gravity-flow column containing 1.0 ml Ni Sepharose High Performance (GE Healthcare, Sweden) pre-equilibrated with buffer *A* containing 10 m*M* imidazole. After 1 h incubation, the column was washed with buffer *A* containing 40 m*M* imidazole. RlmJ was eluted with buffer *A* containing 250 m*M* imidazole, concentrated to a volume of 5.0 ml using a Vivaspin 30 kDa cutoff concentrator (Sartorius Stedim Biotech, Germany) and further purified on a HiLoad 16/60 Superdex 75 column (GE Healthcare, Sweden) pre-equilibrated with buffer *A*. Samples at each purification step were analysed by SDS–PAGE using a PhastSystem (GE Healthcare, Sweden) and 8–25% polyacrylamide gradient PhastGel (GE Healthcare, Sweden). Peak fractions containing RlmJ were concentrated to 11 mg ml^−1^ using a Vivaspin 30 kDa cutoff concentrator.

### 2.2. Crystallization

Initial sitting-drop vapour-diffusion crystallization trials were performed using an Oryx4 crystallization robot (Douglas Instruments, UK). The commercial crystallization screens JCSG+ (Qiagen, Germany), PEG/Ion (Hampton Research, USA) and Structure Screen I and II (Molecular Dimensions Ltd, UK) were used to identify promising crystallization conditions for apo RlmJ. Crystallization drops of 0.6 µl (protein:precipitant ratio of 1:1) were set up in 96-well plates containing 80 µl reservoir solution. Initial hits were obtained after 5 d in condition D1 [0.2 *M* sodium acetate trihydrate, 0.1 *M* Tris–HCl pH 8.5, 30%(*w*/*v*) PEG 4000] of Structure Screen I and II at 293 K in the form of clusters of conjoined thin plate-like crystals. Attempts to optimize the crystallization condition by altering the pH range and PEG concentration or by using the ADDit Additive Screen (Emerald BioSystems, USA) and streak-seeding were not successful. The crystal morphology was improved by replacing the sodium acetate trihydrate with sodium sulfate decahydrate. Single rectangular rod-shaped crystals (30 × 30 × 200 µm) of RlmJ grew within 8 d at 293 K using a reservoir solution consisting of 0.2 *M* sodium sulfate decahydrate, 0.1 *M* Tris–HCl pH 8.5, 30%(*w*/*v*) PEG 4000 and a drop size of 3.0 µl (protein:precipitant ratio of 1:1) equilibrated against 200 µl reservoir solution.

### 2.3. Data collection and processing

For data collection, the crystals were soaked in cryoprotectant (reservoir solution supplemented with 10% glycerol) for 5–10 s. A single crystal was harvested with a litho-loop and vitrified in liquid nitrogen. Diffraction data were collected at 100 K on the ID23-2 microfocus beamline at the European Synchrotron Radiation Facility, Grenoble, France at a wavelength of 0.873 Å. Based on test diffraction images, the data-collection strategy was determined using *EDNA* (Incardona *et al.*, 2009 ▶). To make optimal use of the rectangular rod-shaped crystals, the helical data-collection protocol (Flot *et al.*, 2010 ▶) in the *MxCuBE* program (Gabadinho *et al.*, 2010 ▶) was used during data collection. Diffraction data (180 frames at 1.0° oscillation and 5.0 s exposure time) from the apo RlmJ crystal were collected using a MAR CCD detector. Diffraction images were indexed and integrated using *XDS* (Kabsch, 2010 ▶) and the data were scaled using *XSCALE* (Kabsch, 2010 ▶). Data-collection and processing statistics are summarized in Table 2 ▶.

## 3. Results and discussion

**Figure 1:**
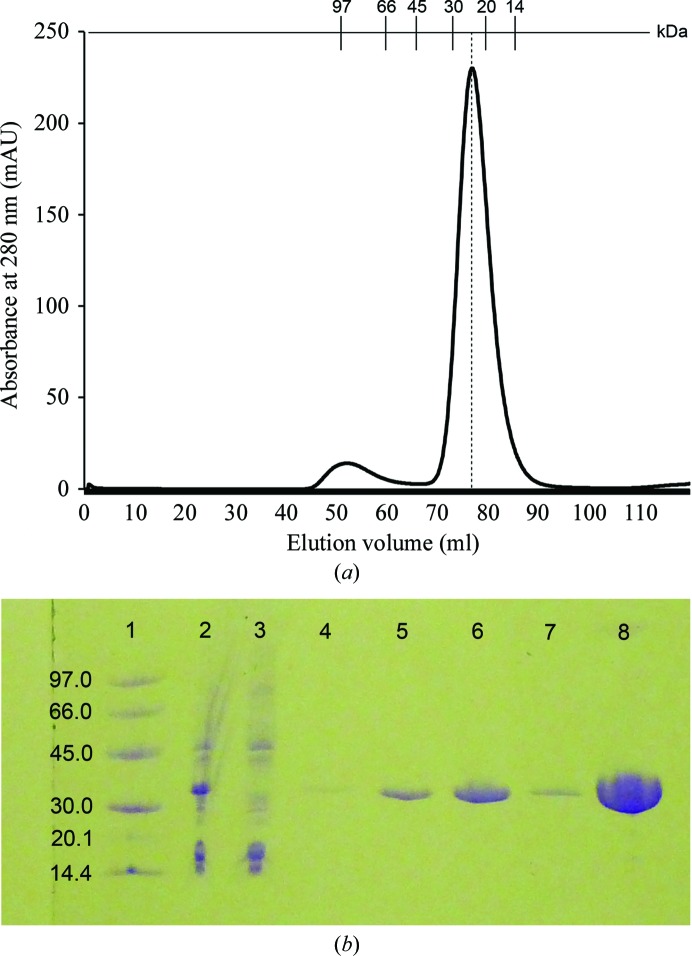
(*a*) Elution profile of RlmJ from a HiLoad 16/60 Superdex 75 gel-filtration column. The major peak corresponds to monomeric RlmJ. Elution volumes of protein molecular-mass standards are indicated at the top. (*b*) PhastGel 8–25% gradient SDS–PAGE analysis of RlmJ purification. Lane 1, molecular-weight marker (labelled in kDa); lane 2, cell lysate; lane 3, flowthrough from Ni Sepharose column; lane 4, final wash fraction from Ni Sepharose column; lanes 5–7, elution fractions from Ni Sepharose column; lane 8, final concentrated RlmJ after HiLoad 16/60 Superdex 75 gel-filtration column.

Recombinant RlmJ was cloned from genomic DNA, overexpressed in *E. coli* and purified to homogeneity using Ni^2+^-affinity chromatography and size-exclusion chromatography (SEC). The SEC elution profile showed a large peak (Fig. 1 ▶ *a*) eluting at an apparent molecular mass of 23.5 kDa. This suggested that recombinant RlmJ with a theoretical molecular mass of 33 kDa is a monomer in solution and folds into a compact globular structure. Analysis of the purified RlmJ by SDS–PAGE and Coomassie staining showed a purity of at least 95% (Fig. 1 ▶ *b*). From a 500 ml culture, 11 mg pure RlmJ was obtained.

**Figure 2:**
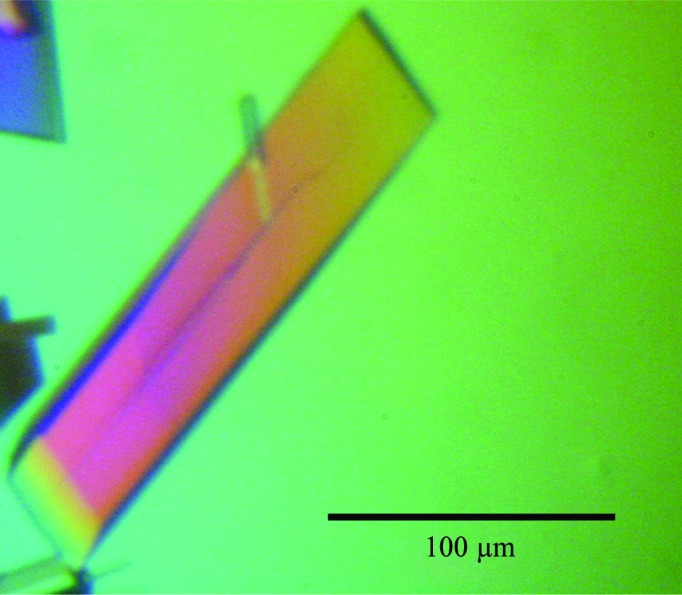
Optimized RlmJ crystals of the type used for data collection.

In initial screens we identified a crystallization condition [0.2 *M* sodium acetate trihydrate, 0.1 *M* Tris–HCl pH 8.5, 30%(*w*/*v*) PEG 4000; Structure Screen I and II, Molecular Dimensions Ltd, UK] in which clusters of conjoined thin plate-like crystals of RlmJ were observed after 5 d at 293 K. Initial attempts to optimize this condition to obtain larger, and more, single crystals failed. Our previous experience with another rRNA MTase from *E. coli*, RlmM, which showed increased thermostability in the presence of sulfate (Punekar *et al.*, 2012 ▶), motivated us to try modifying the initial crystallization condition by replacing sodium acetate trihydrate with sodium sulfate decahydrate. In this optimized crystallization condition, single, rectangular, rod-shaped apo RlmJ crystals of 30 × 30 × 200 µm in size grew within 8 d (Fig. 2 ▶).

**Figure 3:**
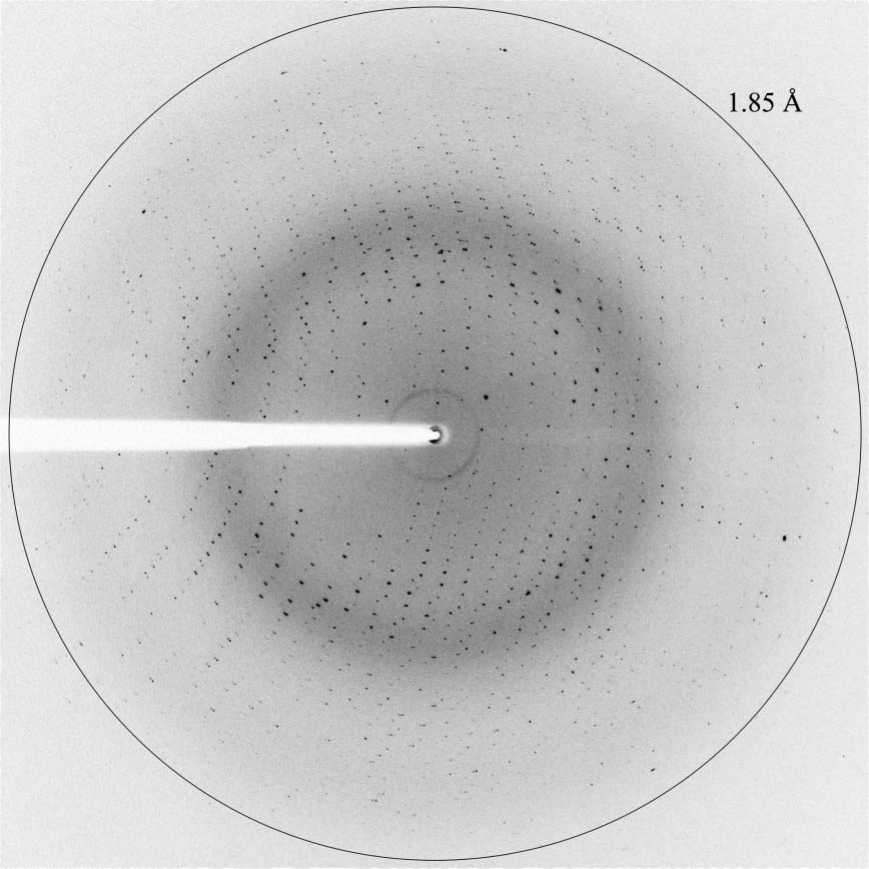
X-ray diffraction pattern from an apo RlmJ crystal. The outer circle indicates a resolution of 1.85 Å.

A complete data set to 1.85 Å resolution (Fig. 3 ▶) was collected from a single crystal using a helical data-collection protocol, which allowed us to use a larger part of the crystal and improved the data quality by minimizing the effect of radiation damage (Flot *et al.*, 2010 ▶). Data-collection and processing statistics are summarized in Table 2 ▶. Preliminary diffraction data analysis using *phenix.xtriage* (Adams *et al.*, 2010 ▶) suggested the presence of two molecules per asymmetric unit, a Matthews coefficient of of 2.20 Å^3^ Da^−1^ and a solvent content of about 44% (Matthews, 1968 ▶). Structure solution of apo RlmJ will be attempted by molecular replacement using the coordinates of the hypothetical LPL1258 protein from *Legionella pneumophila* (PDB entry 2oo3; New York SGX Research Center for Structural Genomics, unpublished work) as a search model. This protein shares 37% sequence identity with RlmJ. The structure of RlmJ will be published elsewhere. Attempts to cocrystallize RlmJ with the cofactor *S*-adenosylmethionine, the cofactor product *S*-­adenosylhomocysteine and substrate analogues are ongoing.

## Figures and Tables

**Table 1:** PCR primer sequences

Forward primer (FP1)	5′-CTTACCGGAACACCTTTACCCATGCTCAGTTATCGCCACA-3′
Reverse primer (RP1)	5′-CAGCAATGGCTGCAATTACTCCGGCACGATCCAG-3′
Forward primer (FP2)	5′-ATGCTCAGTTATCGCCACAGC-3′
Reverse primer (RP2)	5′-GGACTCCGGCACGATCCA-3′

**Table 2:** Data-collection and processing statistics Values in parentheses are for the outermost resolution shell.

No. of crystals	1
Beamline, synchrotron	ID23-2, ESRF, Grenoble, France
Wavelength (Å)	0.873
Detector	MAR CCD
Data-collection temperature (K)	100
Crystal-to-detector distance (mm)	210.5
Rotation range per image (°)	1
Total rotation range (°)	180
Exposure time per image (s)	5
Resolution range (Å)	50.0–1.85 (1.95–1.85)
Space group	*P*2_1_
Unit-cell parameters (Å, °)	*a* = 46.87, *b* = 77.75, *c* = 82.50, α = 90, β = 104, γ = 90
Total No. of measured intensities	184526 (25907)
Unique reflections	48904 (7018)
Completeness (%)	99.6 (98.3)
Multiplicity	3.8 (3.7)
〈*I*/σ(*I*)〉	12.09 (3.36)
*R*_merge_ † (%)	11.9 (60)
*R*_meas_ ‡ (%)	13.9 (67.5)
Overall *B* factor from Wilson plot (Å^2^)	14.6

† *R*_merge_ = 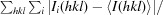 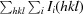.

‡ *R*_meas_ = 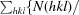 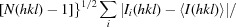 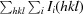.
